# Outcomes of Revision Total Wrist Arthroplasty

**DOI:** 10.5435/JAAOSGlobal-D-21-00035

**Published:** 2021-03-16

**Authors:** Eric R. Wagner, Jason J. Srnec, Michael W. Fort, Laurel A. Barras, Marco Rizzo

**Affiliations:** From the Department of Orthopedic Surgery (Dr. Srnec, Mr. Fort, Dr. Barras, and Dr. Rizzo), Mayo Clinic, Rochester, MN, and the Department of Orthopaedic Surgery (Dr. Wagner), Emory University, Atlanta, GA.; Correspondence to Dr. Rizzo: rizzo.marco@mayo.edu.

## Abstract

In this study, we analyzed the outcomes of revision total wrist arthroplasty done over a 40-year period. During this period, 76 consecutive revision total wrist arthroplasties were done in 69 patients with a mean age of 56 and a follow-up of 10.3 years. Patients commonly experienced pain relief, with 58 (91%) experiencing no or mild pain postoperatively. There was no statistically significant change in flexion or extension, but a statistically significant decrease in ulnar deviation and a corresponding increase in radial deviation. However, a high rate of complications and repeat revision surgery were observed. The primary indications for a repeat revision surgery were distal loosening (n = 11), proximal loosening (n = 1), deformity and pain (n = 8), periprosthetic infection (n = 3), dislocation (n = 2), subluxation (n = 1), intraoperative fracture (n = 1), and suspected metal allergy (n = 1). Complications were distal loosening (n = 15), proximal loosening (n = 3), dislocation (n = 7), intraoperative fracture (n = 7), postoperative fracture (n = 2), and component fracture (n = 3). When advising the patient, clear information must be given about the high rate of complications connected with revision arthroplasty and the risk of further revision, eventually leading to total wrist fusion.

**Level of Evidence:**

IV

Despite the effectiveness of total wrist arthroplasty (TWA) in relieving pain and preserving motion, it has suffered from high rates of complications including instability and implant loosening.^1,2,3,4,5,6,7,8,9,10,11^ In some instances, a revision or salvage procedure is necessary, with most reported revision rates falling in the range of 0% to approximately 20%,^2,5,6,7,8,9,12^ although rates as high as 50% to 60%^4,10^ after TWA have been reported.

In the setting of failed TWA, commonly performed procedures include arthrodesis, resection arthroplasty, and revision TWA. With arthrodesis, poor bone stock can make obtaining fusion difficult.^13,14^ Furthermore, patients lose wrist motion, which potentially compromises extremity function, particularly in the cases of bilateral disease.^15^ With resection arthroplasty, no studies have analyzed the outcome of this procedure. However, experts have suggested resection arthroplasty as a potential salvage option for failed TWA.

Revision TWA represents a motion-sparing option for patients after failed TWA. However, it poses many challenges related to limited and deficient bone stock and soft-tissue stabilizers (Figures 1-3). Very little has been reported examining the outcomes associated with revision TWA. Given this lack of information, the indications for this procedure—and whether it should be a treatment option at all—have yet to be determined. Therefore, the purpose of this study was to assess long-term outcomes of a consecutive series of revision TWAs done over a 40-year period.

**Figure 1:**
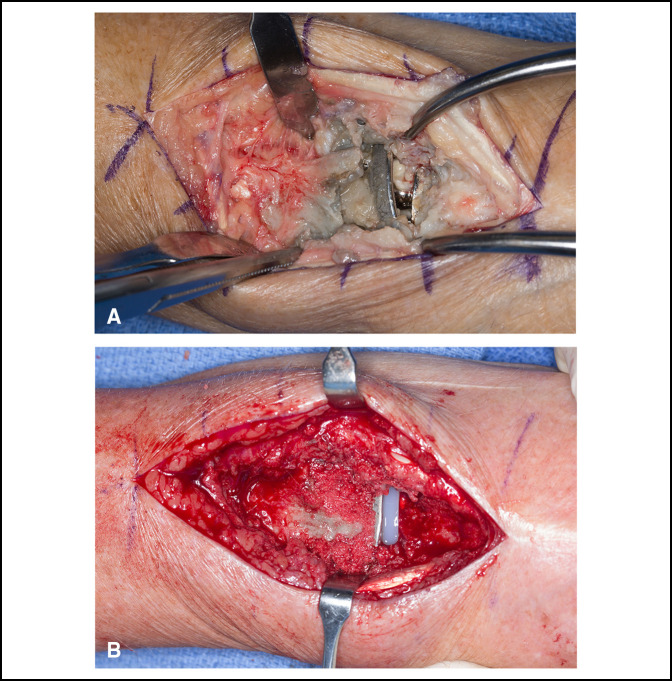
Intraoperative images demonstrating the technically challenging revision total wrist arthroplasty: **A**, failed total wrist arthroplasty with proximal component loosening and **B**, after placement of press-fit radial component, polyethylene spacer, and bone grafting.

**Figure 2:**
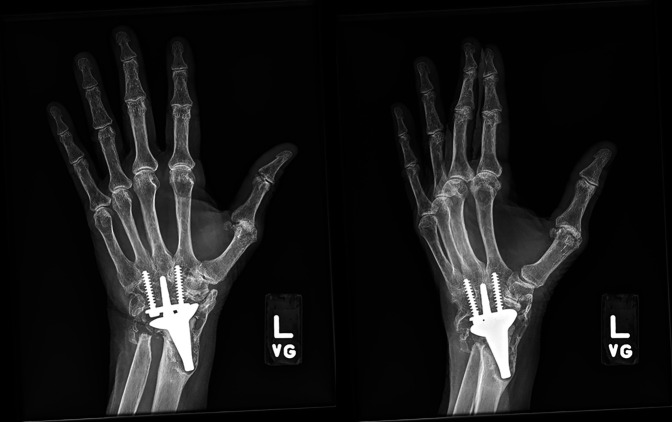
AP and oblique radiographs of the left wrist of a patient with total wrist arthroplasty demonstrating failure and loosening of the proximal component.

**Figure 3:**
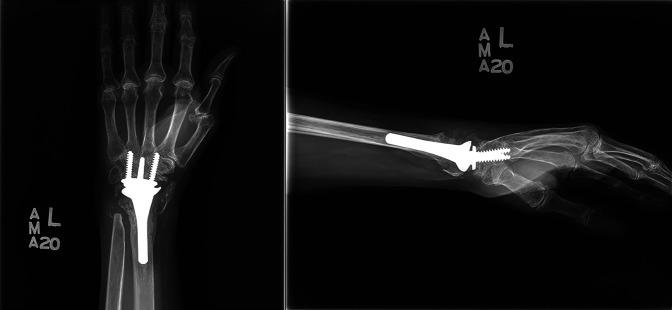
Postoperative AP and lateral radiographs of the left wrist after revision total wrist arthroplasty.

## Methods

After institutional review board approval was obtained, all patients who had undergone revision TWA from 1974 to 2013 were identified. Ten patients were lost to follow-up because of death and were excluded. For the remaining patients, a prospectively collected institutional joint registry^16^ was used to collect patient data, surgical outcomes, and medical history. As part of the joint registry, these variables are collected before and after surgery and at 1, 2, 5, and 10 years, and then every 5 years through questionnaires and interviews by trained staff. Wrist range of motion, indications for revision surgery, and details of fractures and reoperations were not collected in the joint registry and were obtained through reviewing the patients' electronic and paper medical charts. Pain scores were obtained by the physician or trained nonphysician staff preoperatively and at postoperative follow-up and were graded on a scale of none, mild, moderate, or severe. Revision surgery was defined as the removal of any component. Revision surgery was defined as any surgical operation involving the wrist, including revision surgery. A goniometer in the clinic was used to evaluate preoperative and postoperative degrees of range of motion and radial/ulnar deviation. A dynamometer was used to measure grip strength, which was subsequently compared with the contralateral side.

Implant survivorship was analyzed using the Kaplan-Meier model survival curves, and comparisons were done using the Cox proportional hazard regression log-rank test. Preoperative and postoperative outcomes were compared using a paired Student *t* test. Multivariate analysis was not done, given the limited number of outcomes. A *P* value < 0.05 was considered statistically significant.

## Results

Seventy-six revision TWAs were followed for an average of 10.3 years (2.0 to 30.0 years) postoperatively.

### Demographics and Surgical Details

At a mean age of 56 years, 69 patients underwent 76 consecutive revision TWAs from 10 different surgeons over the 40-year period from 1974 to 2013. Bilateral revision TWA was done on seven patients in a staged fashion. Patient demographics and implants used for revision TWA are summarized in Table 1. In this article, the currently available ReMotion (SBI) and Universal (KMI) are considered new generation implants (n = 5, 6.6%), whereas all other implants are considered previous generation. The etiologies of posttraumatic arthritis were distal radius fracture (n = 3), scapholunate advanced collapse (n = 3), lunotriquetral ligament tear (n = 1), scaphotrapeziotrapezoid arthrosis associated with collapse pattern of the wrist (n = 1), history of car accident resulting in unspecified wrist injury treated at outside facility (n = 1), and scaphoid nonunion advanced collapse (n = 1). The primary indications for revision surgery were carpal component loosening (n = 30), radial component loosening (n = 2), pain and wrist deformity (n = 15), dislocation (n = 7), subluxation (n = 7), implant fracture (n = 10), tenosynovitis (n = 2), carpal tunnel syndrome (n = 1), rupture of flexor tendons (n = 1), and extension contracture (n = 1).

**Table 1:**
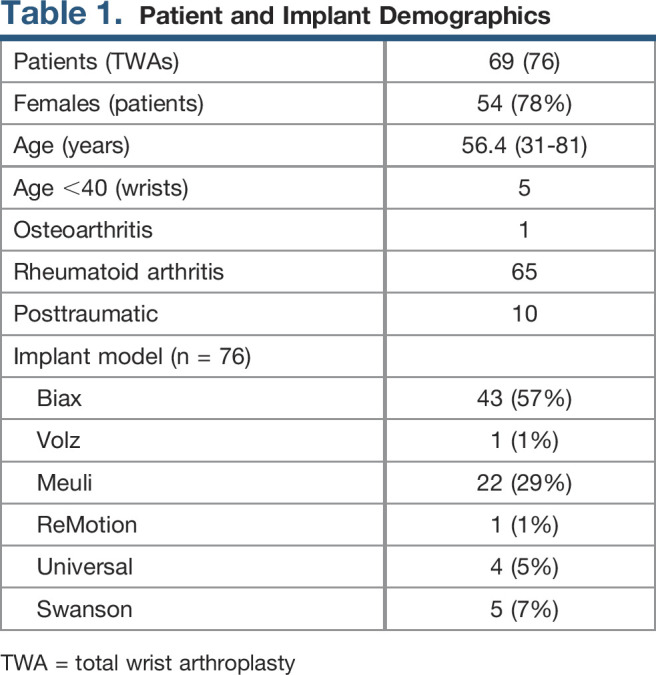
Patient and Implant Demographics

Patients (TWAs)	69 (76)
Females (patients)	54 (78%)
Age (years)	56.4 (31-81)
Age <40 (wrists)	5
Osteoarthritis	1
Rheumatoid arthritis	65
Posttraumatic	10
Implant model (n = 76)	
Biax	43 (57%)
Volz	1 (1%)
Meuli	22 (29%)
ReMotion	1 (1%)
Universal	4 (5%)
Swanson	5 (7%)

TWA = total wrist arthroplasty

### Complications

Seven intraoperative complications were observed, all involving fracture of the index (n = 1), long (n = 2), and ring (n = 1) finger metacarpals; distal radius (n = 1); and radial styloid (n = 2). Both radial styloid fractures were débrided with removal of the broken part of the styloid, and in the case of one long finger metacarpal fracture, the Biaxial medium long-stem component initially used was too large for the canal, which resulted in the fracture. Instead, a small long-stem Biaxial component was used to achieve proper fit. With the distal radius fracture, the Biaxial proximal component was up-sized to bridge the fracture and seat in the portion of intact proximal radius. Postoperatively, the patient was placed in a cast; however, the fracture failed to heal, and approximately 3.5 months later, the patient underwent revision with arthrodesis done. The remaining fractures required no additional interventions. Intraoperative fractures occurred with impaction (n = 3), during removal of the proximal component (n = 2), reaming/broaching in the long metacarpal (n = 1), and during flexion manipulation of a stiff wrist, resulting in radial styloid fracture (n = 1).

Postoperative complications occurred in 26 patients, with 2 (3%) periprosthetic fractures, 15 (20%) aseptic distal loosening, 3 (4%) aseptic proximal loosening, 7 (9%) dislocations, and 3 (4%) component fractures. Four patients had two postoperative complications including distal and proximal loosening (n = 2), postoperative fracture and distal loosening, and component fracture and distal loosening. The postoperative fractures involved the distal radius (n = 1) and carpus/metacarpal (n = 1). The distal radius fracture underwent open reduction and internal fixation with preservation of the prosthesis, whereas the fracture associated with the carpal component was converted to an arthrodesis using a Steinmann pin, which was later removed because of pain. The patient eventually went on to develop a pain-free pseudarthrosis. Implant-specific outcomes are included in Table 2.

**Table 2:**
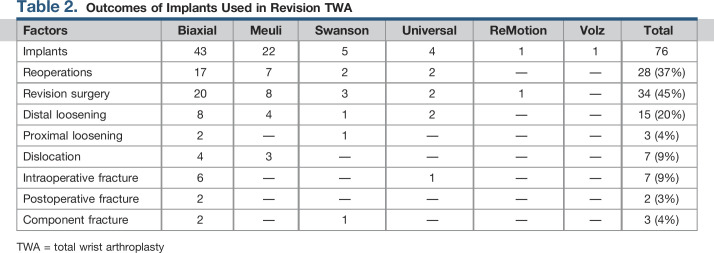
Outcomes of Implants Used in Revision TWA

Factors	Biaxial	Meuli	Swanson	Universal	ReMotion	Volz	Total
Implants	43	22	5	4	1	1	76
Reoperations	17	7	2	2	—	—	28 (37%)
Revision surgery	20	8	3	2	1	—	34 (45%)
Distal loosening	8	4	1	2	—	—	15 (20%)
Proximal loosening	2	—	1	—	—	—	3 (4%)
Dislocation	4	3	—	—	—	—	7 (9%)
Intraoperative fracture	6	—	—	1	—	—	7 (9%)
Postoperative fracture	2	—	—	—	—	—	2 (3%)
Component fracture	2	—	1	—	—	—	3 (4%)

TWA = total wrist arthroplasty

### Repeat Revisions and Reoperations

**Figure 4:**
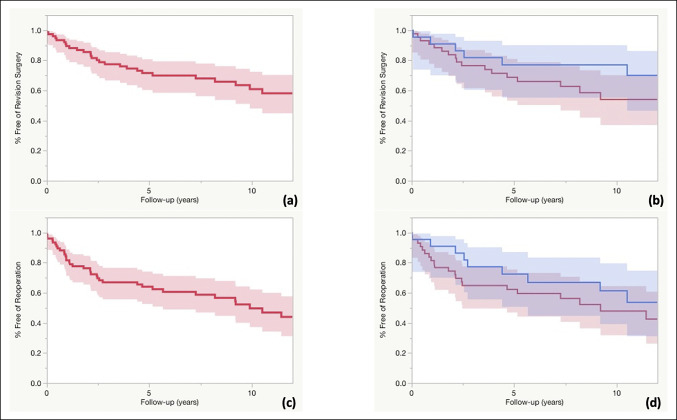
Graph showing survival analyses. **A**, Revision total wrist arthroplasty Kaplan-Meier survival analysis. The 5- and 10-year repeat revision-free survival rates were 71% and 60%, respectively. **B**, The 5- and 10-year repeat revision-free survival rates for biaxial (red) were 68% and 54%, and Meuli (blue) were 77% and 77%, respectively. Error bars represent 95% confidence intervals. **C**, Kaplan-Meier survival analysis showing 5- and 10-year revision surgery-free survival rates of 64% and 49%, respectively. Error bars represent 95% confidence intervals. **D**, The 5- and 10-year revision surgery-free survival rates for biaxial (red) were 62% and 47%, and Meuli (blue) were 72% and 61%, respectively. Error bars represent 95% confidence intervals.

Twenty-eight (37%) implants required repeat revision surgery. The primary indications for a repeat revision surgery were carpal component loosening (n = 11), radial component loosening (n = 1), deformity and pain (n = 8), periprosthetic infection (n = 3), dislocation (n = 2), subluxation (n = 1), intraoperative fracture (n = 1), and suspected metal allergy because of persistent swelling of the dorsum and evidence of osteolysis around the proximal component (n = 1). In the case of intraoperative fracture, casting was initially attempted unsuccessfully and ultimately required a repeat revision procedure undergoing total wrist arthrodesis. The treatments used when the revision surgery failed were repeat revision TWA (n = 15), total wrist arthrodesis (n = 9), resection arthroplasty (n = 3), and resection arthroplasty with fascia lata interposition arthroplasty (n = 1). The Kaplan-Meier survival analysis (Table 3) demonstrated a 5- and 10-year implant survival-free repeat revision surgery (Figure 4A) of 71% and 60%, respectively. Subgroup survival analysis of Biaxial and Meuli implants demonstrated no notable difference in repeat revision-free survival (Figure 4B).

**Table 3:**
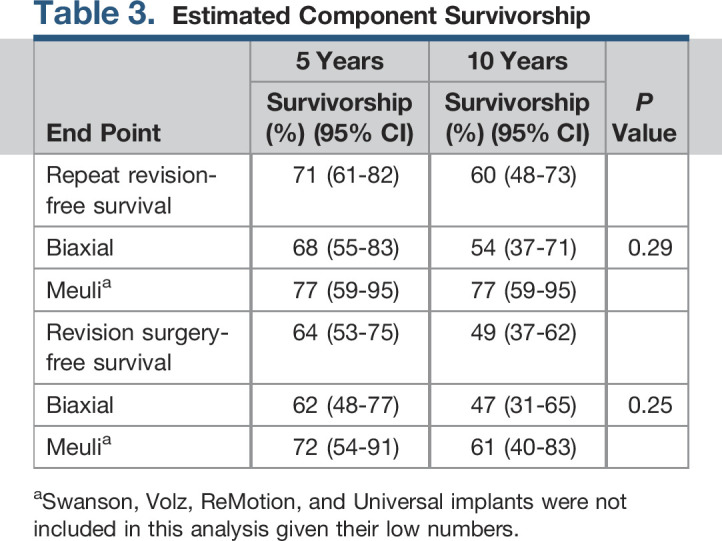
Estimated Component Survivorship

End Point	5 Years	10 Years	*P* Value
	Survivorship (%) (95% CI)	Survivorship (%) (95% CI)	
Repeat revision-free survival	71 (61-82)	60 (48-73)	
Biaxial	68 (55-83)	54 (37-71)	0.29
Meuli^a^	77 (59-95)	77 (59-95)	
Revision surgery-free survival	64 (53-75)	49 (37-62)	
Biaxial	62 (48-77)	47 (31-65)	0.25
Meuli^a^	72 (54-91)	61 (40-83)	

a Swanson, Volz, ReMotion, and Universal implants were not included in this analysis given their low numbers.

In addition to a repeat revision surgery, six reoperations were done including excision of a hamate remnant for recurrent pain (n = 1), heterotopic ossification excision (n = 1), irrigation and débridement for a superficial abscess (n = 1), extensor tendon repair (n = 1), and tenosynovectomies for tendon irritation (n = 2). Five-year and 10-year survival-free revision surgery (Figure 4C) was 64% and 49%, respectively. Subgroup survival analysis of Biaxial and Meuli implants demonstrated no notable difference in reoperation-free survival (Figure 4D).

### Clinical Outcomes

Patients undergoing revision TWA that did not require a repeat revision procedure experienced predictable pain relief, with 91% of patients having no or mild pain postoperatively in comparison with 42% preoperatively (*P* < 0.01). Radial deviation was significantly increased by 5° (*P* = 0.04), and ulnar deviation significantly decreased by 5.2° (*P* = 0.04) postoperatively. No significant difference was observed between preoperative and postoperative grip strength, flexion, or extension in patient undergoing revision TWA that did not require a repeat revision procedure (Table 4).

**Table 4:**
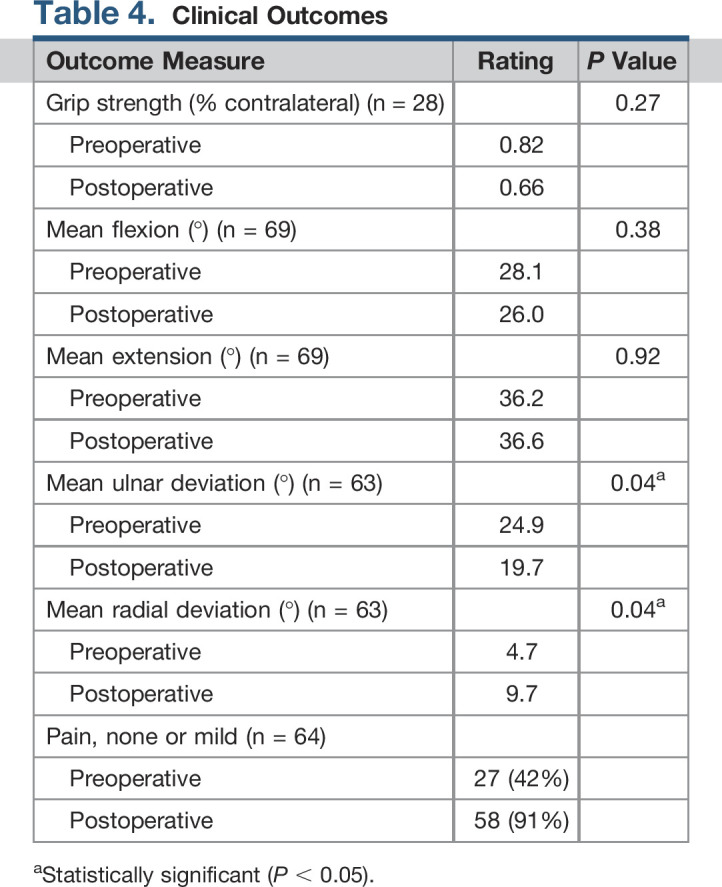
Clinical Outcomes

Outcome Measure	Rating	*P* Value
Grip strength (% contralateral) (n = 28)		0.27
Preoperative	0.82	
Postoperative	0.66	
Mean flexion (°) (n = 69)		0.38
Preoperative	28.1	
Postoperative	26.0	
Mean extension (°) (n = 69)		0.92
Preoperative	36.2	
Postoperative	36.6	
Mean ulnar deviation (°) (n = 63)		0.04^a^
Preoperative	24.9	
Postoperative	19.7	
Mean radial deviation (°) (n = 63)		0.04^a^
Preoperative	4.7	
Postoperative	9.7	
Pain, none or mild (n = 64)		
Preoperative	27 (42%)	
Postoperative	58 (91%)	

a Statistically significant (*P* < 0.05).

## Discussion

Total wrist arthrodesis is commonly done as a salvage procedure for failed TWA, given the challenges associated with revision arthroplasty and its ability to predictably produce a stable and pain-free wrist.^13,14,17^ Despite this, many patients prioritize a motion-sparing alternative to total wrist fusion, such as a revision TWA.^9^ However, very little has been reported pertaining specifically to the results of revision TWA.

Currently, there remain only a few small studies examining the use of a revision arthroplasty to treat a failed previous TWA. In a study of 13 revision TWAs using the standard Biaxial implant, three implants were revised at a follow-up of 31 months for loosening (n = 2) and a circumferential distal radius fracture (n = 1) that occurred intraoperatively when attempting to remove the proximal component.^18^ Four implants (including the implants requiring revision) experienced loosening. Furthermore, five intraoperative complications occurred with longitudinal fracture of the radius (n = 1), perforation of the long-finger metacarpal (n = 3), and a circumferential fracture of the distal radius (n = 1).

Given the difficulties with the Biaxial implant loosening in their previous study, Cobb and Beckenbaugh^19^ attempted the use of a custom long-stemmed multipronged Biaxial implant in revision cases. Short-term results (mean follow-up of 3.8 years) were promising with only two patients requiring revision in a study involving 10 custom long-stemmed multipronged Biaxial implants. Of note, asymptomatic radiolucent lines were found in three of the eight implants that did not require a repeat revision procedure.

In comparison with these previous studies, our series had a higher rate of revision and complications. This can likely be attributed to our longer mean follow-up. However, similar to the finding of Rettig and Beckenbaugh,^18^ the Biaxial implant was associated with a high rate of intraoperative fractures. Interestingly, no intraoperative fractures were noted with the use of the long-stemmed Biaxial implant.^19^ Despite the development of complications, similar to our study, patients commonly achieved satisfaction and pain relief with the procedure.^18,19^ The high rate of complications experienced with revision TWA is likely due to multiple factors. Many patients undergoing revision TWA suffer from rheumatoid arthritis, a disease process that can compromise bone quality and lead to soft-tissue deficiency. Theoretically, this would make implants more susceptible to the development of complications and, in some instances, the need for revision. A further challenge of revision TWA is the bone loss, scar tissue, and altered anatomy that can result from primary TWA.

From a planning and technical perspective, the challenges associated with revision TWA are numerous. It is important to appreciate that revision may not be feasible and that the patients understand that arthrodesis will be necessary. Remaining bone stock and quality of bone are important determinants of the successful revision. Extraction of the failed implant with minimal bone resection is important, and proper surgical exposure is necessary. Meticulous technique and patience with the extraction process will help preserve bone and minimize complications such as fractures. Cases of previously cemented primary implants will take more bone with removal. Use of flexible osteotomes and sequential k-wires to circumferentially break the bone-implant or bone-cement interfaces are helpful. After implant removal, assessing the residual bone quality and soft tissues will help determine whether a revision is possible. Sending tissue for analysis with pathology (and, at times, microbiology) is routine to ensure that there is no underlying infection. In addition, analysis of the resected implant in the microbiology laboratory is routinely done. More often, the bone will be the main determinant, but excessive laxity or tightness of the stabilizing soft tissues has the potential to limit our options. If revision is deemed feasible, meticulous technique will again be essential. The use of impaction bone-grafting can help fill in bone voids. Allograft bone grafting is more often used. Because of the previously mentioned challenges in removing, cementing is less preferred than impaction grafting but may be necessary. Although not always an option, the use of custom implants with longer and/or wider stems/components are helpful. In many cases of revision, aftercare is modified. Longer postoperative periods of immobilization are often used to help bone graft incorporate and optimize stability.

The limitations of our findings should be considered. Our investigation was retrospective in nature and is therefore limited to the data that were previously collected. The absence of validated patient-reported outcomes measured in the original data is an important weakness. Furthermore, given the long duration of this study, some of the implants have been discontinued or had their design modified. In addition, this is a single-institution study, inherently creating a referral bias. However, a strength of our study is the utilization of a large consecutive series of revision TWAs and a prospectively collected total joint registry that used standardized follow-up and collection of outcomes.

Overall, the high rate of complications observed in this mostly historical cohort highlights the difficulty of performing a revision TWA. Given the poor bone stock, soft-tissue deficiency, and scar tissue from the primary TWA, the revision implant is prone to instability, component loosening, and other complications. These results should serve as a valuable reference for those considering revision arthroplasty and demonstrate the need for improved techniques and implants. Until such innovations are able to improve on our results, caution should be used when considering a revision TWA. Furthermore, as has been preliminarily started in primary TWA,^20^ there is a critical need for standardized data collection with the use of a national arthroplasty registry.
